# Effects of irisin on inflammatory and apoptotic markers in the Caco-2 colon cancer cell line

**DOI:** 10.1186/s12860-026-00568-w

**Published:** 2026-01-22

**Authors:** Elif Zeynep Ozturk, Ebubekir Bakan, Nurcan Kilic Baygutalp, Zafer Bayraktutan

**Affiliations:** 1 Department of Fundamental Sciences, Faculty of Engineering and Natural Sciences, Kutahya Health Sciences University, Kutahya, 43100 Turkey; 2 Department of Medical Biochemistry, Faculty of Medicine, Kars Kafkas University, Kars, 36100 Turkey; 3 Department of Biochemistry, Faculty of Pharmacy, Ataturk University, Erzurum, 25240 Turkey; 4 Department of Medical Biochemistry, Faculty of Medicine, Ataturk University, Erzurum, 25240 Turkey

**Keywords:** Apoptosis, Colon cancer, Inflammation, Irisin, NF –κB pathway

## Abstract

**Objective:**

Irisin is a myokine that is secreted by muscle tissue and is recognized for its regulatory effects on inflammation. The objective of this study was to examine the potential anti-inflammatory function of irisin in the context of colon cancer, with a focus on its effects on the Caco-2 cell line. Method: Specifically, pro-inflammatory cytokine levels (IL-6 and TNF-α), apoptotic marker Caspase-3 activity, and changes in the NF-κB signaling pathway were assessed using ELISA assays.

**Results:**

The findings of this study indicated a substantial augmentation in IL-6 levels in the 100 nM irisin-treated group in comparison to the control group (*p* < 0.05). Furthermore, a significant divergence in Caspase-3 activity was observed between the 10 nM and 100 nM irisin groups (*p* < 0.05). While direct antiproliferative effects of irisin on Caco-2 cells were not evident in analyses of cellular toxicity, a significant increase in Caspase-3 activity suggested activation of apoptotic pathways. Furthermore, the upregulation of NF-κB signaling, which is generally linked to cancer progression through the suppression of apoptosis, was observed following irisin treatment.

**Conclusion:**

Irisin did not exert a consistent antiproliferative effect in Caco-2 cells under the tested conditions. Instead, irisin exposure was associated with alterations in inflammatory and apoptotic markers, including increased IL-6 and NF-κB levels and modulation of caspase-3 activity. These findings suggest that irisin may act as a context-dependent regulator of inflammatory and apoptotic signaling rather than a direct anti-inflammatory or antiproliferative agent in colon cancer cells.

## Introduction

Adipose tissue in the human body can be categorized into two distinct types: white and brown. White adipose tissue fulfills the primary function of an energy reservoir, storing excess energy in the form of triglycerides. In addition, it functions as an endocrine organ that contributes to energy homeostasis. Conversely, brown adipose tissue (BAT) is imperative for thermoregulatory processes, functioning by expending energy as heat [1]. Regular exercise is widely recognized for its beneficial effects on metabolism, both in health and disease [2]. Studies have indicated that a higher ratio of brown adipose tissue correlates with a lower body mass index (BMI), whereas an increase in white adipose tissue is associated with a heightened risk of insulin resistance, obesity, and Type 2 Diabetes [2].

Skeletal muscle has also been identified as an endocrine organ capable of producing and secreting numerous paracrine and endocrine myokines [3, 4]. Among these, irisin, predominantly secreted by skeletal muscle during physical exercise, is known for its thermogenic activity through the conversion of white adipose tissue to brown adipose tissue. Irisin has been demonstrated to induce substantial alterations in subcutaneous white adipose tissue, with a concomitant enhancement in the expression of uncoupling protein 1 (UCP1). This, in turn, has been shown to promote the process of thermogenesis in newly formed brown adipose tissue [5]. Consequently, irisin contributes to increased total body energy expenditure and reduced obesity-induced insulin resistance. A multitude of studies have been conducted to investigate the correlation between irisin and glucose metabolism. These studies propose that irisin may serve as a therapeutic molecule, with the potential to address insulin resistance in obesity and Type 2 diabetes [6].

In addition, physical exercise is acknowledged for its protective effects against cancer. It is recognized to improve cancer prognosis, although the precise mechanisms remain incompletely understood [7]. Regular physical activity has been shown to reduce the risk of colorectal cancer and to improve clinical outcomes, effects that are partly attributed to exercise-induced molecular adaptations. Among the proposed mediators linking exercise to cancer-related biological effects, irisin has emerged as a key muscle-derived myokine released into the circulation during physical activity. Irisin has been reported to regulate metabolic homeostasis and inflammatory signaling and to modulate cellular stress responses in various tissues.

Given the established association between exercise and colorectal cancer biology, it is plausible that irisin may contribute to the regulation of inflammatory and apoptotic pathways in colon cancer cells. However, irisin is not a classical chemokine or a direct apoptosis-inducing factor, and its role in colon cancer–related cellular signaling remains incompletely understood. Therefore, the present study aimed to investigate the effects of irisin on selected inflammatory and apoptotic markers in Caco-2 cells as an in vitro model, in order to explore a potential molecular link between exercise and colon cancer–related cellular responses, rather than to directly induce apoptosis. Recent studies have indicated that irisin may play a protective role in various types of cancer, including breast, lung, pancreatic, renal, and colorectal cancers. This suggests that irisin could serve as a biomarker and a potential therapeutic target [8, 9].

Maalouf and El Khoury proposed that irisin exerts an indirect effect on cancer risk reduction and improved prognosis by promoting the browning of white adipose tissue (transformation into beige adipose tissue). This process leads to a reduction in the secretion of pro-inflammatory cytokines. In contrast, other studies have proposed a direct role for irisin in the inhibition of cancer cell proliferation, invasion, and metastasis [10, 11]. However, the direct mechanisms of irisin in cancer remain to be elucidated and require further investigation.

In this study, the antiproliferative effects of irisin on the colon cancer cell line Caco-2 were evaluated using the Cell Counting Kit-8 (CVDK-8) assay. The study also examined the apoptotic contributions of irisin, which were determined via Caspase-3 levels. Furthermore, the indirect antineoplastic effects of irisin were assessed by measuring key inflammatory mediators, such as TNF-α, IL-6, and NF-κB. The objective of this study was to elucidate the direct and indirect potential mechanisms of irisin and its prospective contributions to colon cancer treatment strategies.

## Materials and methods

### Cell culture

The human colorectal adenocarcinoma cell line Caco-2 was used in this study. Cells were obtained from the American Type Culture Collection (ATCC, Manassas, VA, USA; catalog no. HTB-37). The cells were grown in Dulbecco’s Modified Eagle’s Medium (Biowest, USA) with 10% (vol/vol) fetal bovine serum. All cells were incubated at 37 °C in an atmosphere of 5% CO2 in the air, and sub-cultured beyond 60–70% confluency.

#### Mycoplasma testing

Cells were routinely tested for mycoplasma contamination using a PCR-based assay, and all experiments were performed with mycoplasma-negative cultures.

#### Authentication

The identity of the Caco-2 cell line was confirmed by short tandem repeat (STR) profiling provided by the supplier.

#### Source and catalog number

Caco-2 cells were purchased from ATCC (catalog no. HTB-37).

#### Passage number

Cells between passages 10 and 20 were used for all experiments.

#### Culture conditions

Cells were maintained in Dulbecco’s Modified Eagle Medium (DMEM) supplemented with 10% fetal bovine serum (FBS) and 1% penicillin/streptomycin at 37 °C in a humidified atmosphere containing 5% CO₂.

#### Good cell culture practice

All cell culture procedures were performed according to international guidelines on good cell culture practice (GCCP).

#### Cross-contamination check

The Caco-2 cell line was cross-checked against the International Cell Line Authentication Committee (ICLAC) and ExPASy Cellosaurus databases. No records of misidentification or cross-contamination were found.

The concentrations of irisin used in this study were selected based on previously published in vitro literature. In particular, Moon and Mantzoros (2014) defined irisin concentrations of 5–10 nM as physiologically relevant and 50–100 nM as pharmacological in cancer cell line models, including colon cancer cells. Based on this classification, 10 nM was chosen as a physiological concentration, while 100 nM was used as a pharmacological dose to assess potential dose-dependent effects under in vitro conditions.

### Chemicals

Human recombinant irisin (Catalog no: BITP3212) was purchased from Apollo Scientific (UK), Cell Viability Detection Kit − 8 (CVDK − 8) from Eco-Tech (TR). IL-6 ELISA kit (Cat. No: E0090Hu) was obtained from BT -Lab (Korea), TNF- α ELISA kit (Cat. No: E0082Hu) from BT -Lab (Korea), NF-κB ELISA kit (Cat. No: E3048Hu) from BT -Lab (Korea), and caspase − 3 ELISA kit (Cat. No: E4804 Hu) from BT -Lab (Korea).

### Proliferation assay

Cells were treated with irisin for 48 h, and then a CVDK − 8 kit was used for proliferation measurement. In the evaluation of proliferation and viability, 10% CVDK solution was added to the wells according to the CVDK − 8 kit protocol [12]. Dehydrogenases reduce CVDK in cells to give an orange-colored product (formazan). It was incubated for 2 h, and the intensity of the color formed was determined with an ELISA reader at a wavelength of 450 nm. Only cells grown in a growth medium were used as a negative control, and 5-fluorouracil (5-FU) at 500 µM concentration was used as a positive control. The proliferation test was performed on cells with 4 different doses of irisin: Group 1: 5nM irisin, Group 2: 10 nM irisin, Group 3: 50 nM irisin, and Group 4: 100 nM irisin, each of the irisin concentrations was added to each respective group.

### Biochemical analyses

Physiological (10 nM) and pharmacological doses (100 nM) were examined for ELISA tests. After 48 h of incubation with irisin, IL-6, caspase − 3, TNF-α, and NF -κB were analysed according to the kit procedure and measured in a multiple spectrophotometer device. All experiments were performed with duplicate samples and repeated three times.

### Statistical analysis

Statistical analyses were performed with the SPSS 23.0 package program for Windows. The conformity of the data to the normal distribution was evaluated with the Shapiro-Wilk test. Student’s t-test was used to compare the irisin-administered and control groups for cytotoxicity evaluation. A one-way ANOVA test was used to evaluate the ELISA results and to compare the groups, post hoch analysis was done with the Tukey test. *p* < 0.05 values were considered statistically significant.

## Results

### Regulation of cell proliferation by irisin in Caco-2 cell lines

**Fig. 1:**
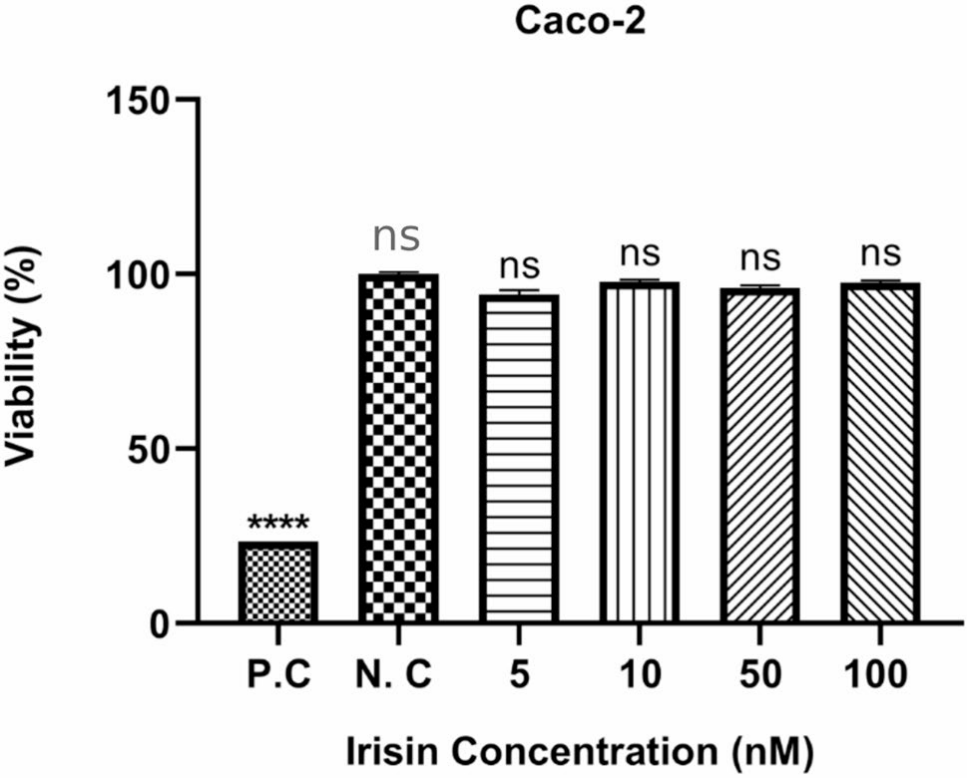
Regulation of cell proliferation by irisin in Caco − 2 cell lines. The cell culture was performed as described in detail in the Methods section. The cells were treated with irisin at indicated concentrations for 48 h, and cell proliferation assay was then performed as described in detail in the Methods section. Proliferation data were analysed using student-t test. Values are means (*n* = 3). 5-fluorouracil (5 -FU) 500 µg/ml for 48 h was used for a positive control

The proliferation rates resulting from the 48-hour cytotoxicity test (CVDK − 8) are displayed in Fig. 1. Cell viability did not differ significantly between the control group and any of the irisin-treated groups (*p* > 0.05). Although a statistically significant change was observed at 5 nM, higher irisin concentrations did not produce a consistent dose-dependent effect. Our observations revealed that irisin does not regulate cell proliferation in a dose-dependent manner in human colon cancer cell lines.

**Fig. 2:**
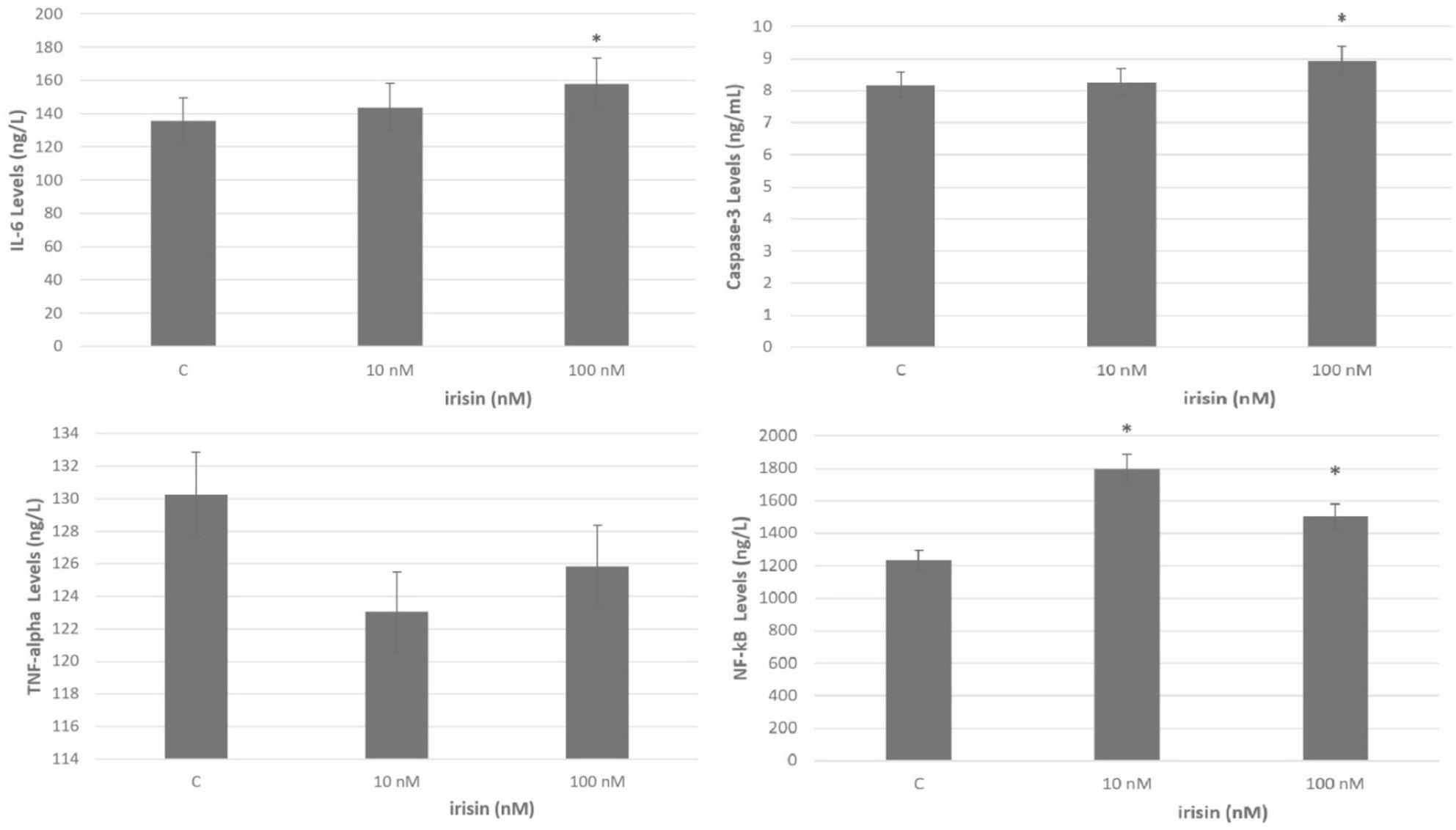
Graph of the mean IL-6, caspase-3, TNF-α and NF-κB of irisin-treated (10–100 nM) Caco-2 cells and control group

Figure 2; Table 1 show the IL -6, caspase − 3, TNF -α, and NF -kB levels of Caco − 2 cells treated with 10 nM and 100 nM irisin and control cells not treated with irisin. There was no difference in IL -6 levels between the group to which 10 nM irisin was applied and the control group (*p* > 0.05). The difference between the 100 nM irisin-applied group and the control group was significant (*p* < 0.05).

An examination of the Caspase-3 levels in Caco-2 cells treated with 10 nM irisin reveals that there is no statistically significant difference between the irisin-treated group and the control group (*p* > 0.05). However, a significant disparity in caspase-3 levels was observed between the 100nM irisin-treated group and both the control and 10nM irisin-treated groups (*p* < 0.05). TNF-α levels did not demonstrate a significant difference between the irisin-treated and control groups (*p* > 0.05).

**Table 1:** Effect of different Irisin concentrations (10–100 nM) on IL -6, TNF -α, caspase − 3 and NF -kB levels in Caco − 2 cell line. A: statistically significant difference at the *P* < 0.05 level between the control and 10 (nM) Irisin groups. B: statistically significant difference at the *P* < 0.05 level between the control and 100 (nM) Irisin groups. C: 10 (nM) and 100 statistically significant difference at *P* < 0.05 level between (nM) Irisin groups

Groups	IL -6 Levels (Mean (ng/l) ± SD)	Groups	TNF-α Levels (Mean (ng/l) ± SD)
10-nM irisin	143.77 ± 10.97	10-nM irisin	123.04 ± 6.09
100-nM irisin	157.68 ± 7.65^b^	100-nM irisin	125.85 ± 6.81
Control	135.83 ± 7.86	Control	130.25 ± 10.35
	**Caspase − 3 Levels** **(Mean (ng/l) ± SD)**		**NF -κB Levels** **(Mean (ng/l) ± SD)**
10-nM irisin	8.26 ± 0.15^c^	10-nM irisin	1797.18 ± 35.54^c^
100-nM irisin	8.95 ± 0.38^b^	100-nM irisin	1505.98 ± 49.99^b^
Control	8.19 ± 0.16	Control	1236.06 ± 75.51^a^

## Discussion

Recent studies have focused on the relationship between exercise and cancer [13], and numerous reports suggest a link between irisin and cancer [14]. Exerkines, including irisin, are signaling molecules released in response to exercise, exerting their biological effects through endocrine, paracrine, or autocrine pathways [15]. To date, approximately 2,000 exerkines have been identified, with irisin prominently regulating various metabolic processes [16]. Irisin has been demonstrated to inhibit pancreatic cancer cell growth via the AMPK-mTOR pathway, highlighting its potential as a chemotherapeutic agent [17].

In the present study, irisin did not prevent cell proliferation; rather, it altered cytokine (IL-6), transcription factor (NF-κB), and apoptotic factor (caspase-3) levels. Caspase-3, a well-established marker of apoptosis [18], has been shown to increase in levels concomitant with the activation of apoptotic mechanisms. However, the concomitant increase in NF-κB, which plays a significant role in inflammation and carcinogenesis, may hinder apoptosis by enhancing anti-apoptotic mechanisms and promoting cellular proliferation [19].

The simultaneous increase in caspase-3 activity and NF-κB signaling introduces an important interpretative consideration. NF-κB activation may reflect a compensatory pro-survival response induced by apoptotic stress, as NF-κB is known to regulate cell survival pathways under stress conditions. Alternatively, NF-κB activation may be part of an inflammatory feedback mechanism, particularly in association with increased IL-6 expression. Given the lack of time-dependent analyses or targeted pathway inhibition, it is not possible to determine whether NF-κB activation is a downstream consequence of apoptosis or an upstream regulator of inflammatory signaling. Further mechanistic studies are required to clarify this relationship.

The positive effects of irisin on glucose metabolism suggest a potential role in obesity-related cancers, thereby stimulating further exploration of its association with cancer. A paucity of studies has been conducted on the effects of irisin on colon cancer. In contrast, Moon et al. [20] reported no significant anticancer effect of irisin in colon cancer cell lines. Conversely, our study, employing the Caco-2 cell line, did not detect direct antiproliferative activity. However, we explored indirect effects by examining cytokine levels, a parameter not evaluated in Moon et al.‘s research. Further studies have identified increased irisin expression in colon cancer tissues and cachexia-associated tumors, indicating a complex association between irisin and cancer [21, 22].

Interactions between IL-6 and TNF-α have been demonstrated to amplify inflammatory responses, thereby facilitating the infiltration of inflammatory cells into tissues [23]. Current cancer research increasingly addresses obesity, the tumor microenvironment, and inflammation [24], emphasizing the therapeutic potential of exercise. It has been reported that exercise has the capacity to normalize elevated TNF-α levels, thereby contributing to anticancer effects by reducing inflammation [25]. In the present study, TNF-α levels exhibited a downward trend following irisin treatment. However, this difference did not attain statistical significance (*p* > 0.05), indicating that the direct effects of irisin on TNF-α are limited in vitro. Nonetheless, these effects may manifest differently in vivo.

The results of this study indicate the presence of activated apoptotic mechanisms, as evidenced by an increase in caspase-3, yet inadequate antiproliferative effects resulting from persistent inflammation, as indicated by elevated levels of IL-6 and NF-κB. NF-κB activation has been demonstrated to promote inflammation and to activate anti-apoptotic pathways. While irisin has demonstrated therapeutic efficacy in various cancers, including lung [10], breast [26], prostate [14], hepatocellular carcinoma [27], osteosarcoma [11], and pancreatic cancer [17], antiproliferative effects were absent in colon cancer studies, including our own and that of Moon et al. [28]. This discrepancy may be attributable to the presence of distinct mechanistic pathways in colon cancer. Furthermore, Şekerci et al. reported that Meteorin-like protein at a dose of 200 ng exhibited a cytotoxic effect on Caco-2 cells, suggesting that a similar effect threshold may exist for irisin. Indeed, irisin may also demonstrate a cytotoxic effect at concentrations above 100 ng [29]. Further research in vitro, in vivo, and in clinical settings is necessary to achieve a more comprehensive understanding.

This study uniquely investigates the antiproliferative and anti-inflammatory effects of irisin on Caco-2 cells, thereby complementing prior research. Although the findings of this study are consistent with those of Moon et al., which demonstrated minimal antiproliferative effects, the significant activation of caspase-3 indicates a potential apoptotic influence of irisin. It is conceivable that elevated irisin concentrations could be indicative of antiproliferative properties. The present findings underscore the importance of dosage-dependent variability in irisin’s effectiveness, thus providing significant insights for future therapeutic applications.

The results of the present study indicate the activation of apoptotic mechanisms, as evidenced by increased caspase-3 levels, despite the absence of significant antiproliferative effects in the Caco-2 cell line. This finding may be explained by the persistence of inflammatory signaling, particularly through elevated IL-6 and NF-κB levels, which are known to promote inflammation and activate anti-apoptotic pathways. Although irisin has demonstrated therapeutic or antiproliferative effects in several cancer types, including lung, breast, prostate, hepatocellular carcinoma, osteosarcoma, and pancreatic cancer, similar effects have not been consistently observed in colon cancer models. Both our findings and those reported by Moon et al. suggest that colon cancer may involve distinct mechanistic pathways that limit the direct antiproliferative efficacy of irisin. Moreover, previous studies have indicated that related exerkines may exhibit cytotoxic effects at higher concentrations, suggesting the presence of a potential dose-dependent threshold for antiproliferative activity in colon cancer cells.

Despite these findings, several limitations should be acknowledged. The absence of mechanistic validation using pathway inhibition or gene knockdown approaches prevents definitive conclusions regarding whether the observed NF-κB activation represents a causal signaling event or a compensatory cellular response to apoptotic stress. Additionally, the lack of time-dependent analyses limits the interpretation of dynamic signaling interactions among NF-κB, IL-6, and caspase-3. Another limitation is the exclusive use of the Caco-2 cell line, which represents a differentiated enterocyte-like phenotype rather than a highly proliferative colorectal adenocarcinoma model, thereby restricting the generalizability of the findings to other colorectal cancer subtypes. These limitations highlight the need for future studies incorporating targeted modulation of NF-κB signaling, broader dose–response and time-course analyses, additional colorectal cancer cell lines, and in vivo models to further elucidate the mechanistic and therapeutic relevance of irisin in colon cancer.

## Conclusions

In conclusion, irisin exposure did not induce a significant antiproliferative effect in Caco-2 cells under the conditions tested. However, irisin modulated key inflammatory and apoptotic markers, including IL-6, NF-κB, and caspase-3, suggesting context-dependent cellular responses. These findings provide a basis for future mechanistic studies to clarify the role of irisin in colon cancer biology.

## Data Availability

All data generated or analysed during this study are included in this published article.

## Declarations

### Preprint statement

This manuscript has previously been posted as a preprint on the Research Square platform (DOI: 10.21203/rs.3.rs-6890787/v1).
